# The Role of Glutathione in Protecting against the Severe Inflammatory Response Triggered by COVID-19

**DOI:** 10.3390/antiox9070624

**Published:** 2020-07-16

**Authors:** Francesca Silvagno, Annamaria Vernone, Gian Piero Pescarmona

**Affiliations:** Department of Oncology, University of Torino, Via Santena 5 bis, 10126 Torino, Italy; annamaria.vernone@unito.it (A.V.); gianpiero.pescarmona@unito.it (G.P.P.)

**Keywords:** SARS-CoV-2, angiotensin-converting enzyme (ACE), angiotensin-converting enzyme 2 (ACE2), glutathione, inflammation, ROS, *N*-acetylcysteine, glycine, chloroquine, paracetamol

## Abstract

The novel COVID-19 pandemic is affecting the world’s population differently: mostly in the presence of conditions such as aging, diabetes and hypertension the virus triggers a lethal cytokine storm and patients die from acute respiratory distress syndrome, whereas in many cases the disease has a mild or even asymptomatic progression. A common denominator in all conditions associated with COVID-19 appears to be the impaired redox homeostasis responsible for reactive oxygen species (ROS) accumulation; therefore, levels of glutathione (GSH), the key anti-oxidant guardian in all tissues, could be critical in extinguishing the exacerbated inflammation that triggers organ failure in COVID-19. The present review provides a biochemical investigation of the mechanisms leading to deadly inflammation in severe COVID-19, counterbalanced by GSH. The pathways competing for GSH are described to illustrate the events concurring to cause a depletion of endogenous GSH stocks. Drawing on evidence from literature that demonstrates the reduced levels of GSH in the main conditions clinically associated with severe disease, we highlight the relevance of restoring GSH levels in the attempt to protect the most vulnerable subjects from severe symptoms of COVID-19. Finally, we discuss the current data about the feasibility of increasing GSH levels, which could be used to prevent and subdue the disease.

## 1. Introduction

Lung inflammation is the main cause of life-threatening respiratory disorders at the severe stage of SARS-CoV-2 infection, characterized by the so-called “cytokine release syndrome (CRS)”.

The key to fighting this harmful inflammatory response resides in: (i) addressing the mechanism of the virus penetration into the cell, mediated by binding to and inactivation of the ACE2 protein; (ii) contrasting the exacerbation of the inflammatory response. The standard pharmacological approach would suggest either the use of an antiviral drug with the aim of blocking viral replication or the exploitation of drugs previously validated as inhibitors of some inflammatory pathway in other chronic diseases. Unfortunately, these drugs are ineffective in healing the most severe cases of SARS-CoV-2, and additionally, they have several side effects. The baffling aspect of this disease is the great heterogeneity of response among patients, ranging from severe symptoms to asymptomatic progression. Understanding the protective mechanisms and the reasons of their failure could provide a breakthrough in the quest for a cure.

The inflammatory response can be traced back to the pathway of viral entry through its receptor ACE2. Angiotensin-converting enzyme 2 (ACE2) is a protease that, with its companion the angiotensin-converting enzyme ACE, takes part in the renin-angiotensin system (RAS). They are localized at the cell surface and compete for the same substrates, angiotensin I and II. ACE2 counters the activity of ACE by reducing the amount of angiotensin-II (ANGII) and increasing ang (1-7) peptide. The downstream effects of the two enzymes are opposite: ACE activity leads to vasoconstriction, oxidative stress, inflammation and apoptosis, whereas ACE2 causes vasodilatation, angiogenesis and anti-inflammatory, anti-oxidative and anti-apoptotic effects [1]. The oxidative stress generated by ACE activity is due to the effects of its product, ANGII, which increases the production of reactive oxygen species (ROS) through the activation of NADPH oxidase and the generation of peroxynitrite anions. In contrast, the ang (1-7) peptide synthesized by ACE2 activity leads to a downregulation of pro-oxidant pathways, which prevents or attenuates the cellular damage induced by oxidative stress.

**Figure 1:**
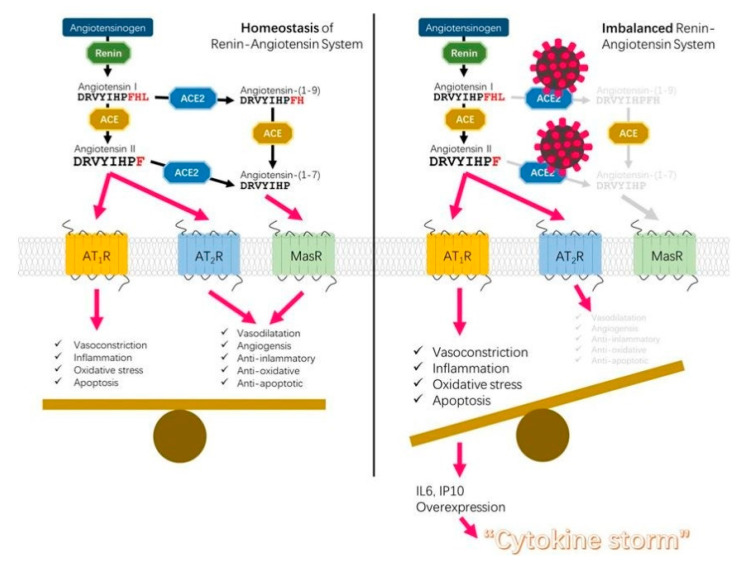
A comprehensive scheme of the interactions between the molecules involved in the renin-angiotensin system (RAS) from [3], copyright 2020 by Gang Niu. Reprinted with permission.

Each person has a different balance between ACE and ACE2 and can be more prone to inflammation if ACE prevails. When this happens, and additionally infection by SARS-CoV-2 downregulates ACE2 abundance on cell surfaces, as suggested by evidence from related coronaviruses [2], the result is the toxic overaccumulation of ANGII, exacerbated inflammation and, finally, acute respiratory distress syndrome and fulminant myocarditis (Figure 1). A different balance of ACE/ACE2 can explain the heterogeneous responses to infection caused by the same virus. The link between the dysregulation of the RAS cascade and the likelihood or severity of SARS-CoV-2 infection has been discussed in some recent works [3,4], and it is a matter of importance when the effects of the RAS inhibitors are debated [5].

Reducing the oxidative stress secondary to the imbalance between ACE and ACE2 could be the best approach for the prevention and treatment of COVID-19. Oxidative stress constitutes a failure of anti-oxidation defense systems to keep ROS and reactive nitrogen species in check. ROS are signaling molecules that induce the release of pro-inflammatory cytokines [6], and the dysregulation of this response plays an essential role in the development of inflammation [7].

Glutathione (GSH) has the function of “master antioxidant” in all tissues; the high concentration of the reduced form (millimolar) highlights its central role in the control of many processes such as detoxification, protein folding, antiviral defense and immune response [8].

The aim of this review is to provide a brief overview of the protective action of GSH against the exacerbated inflammation triggered by COVID-19 upon ACE/ACE2 imbalance. Furthermore, we discuss the evidence of the low levels of GSH found in the conditions associated with the severe outcome of the disease, with the intension of looking into the relevance of restoring GSH levels in the attempt to protect the most vulnerable subjects from COVID-19.

## 2. Cellular GSH Homeostasis

Glutathione, a tripeptide composed of glutamate, cysteine and glycine, is an antioxidant molecule ubiquitous in most living organisms. Intracellular GSH balance is maintained by de novo synthesis, regeneration from the oxidized form, GSSG, and extracellular GSH uptake. In transporting epithelial cells, such as enterocytes, γ-glutamyl transferase (γ-GT) and dipeptidase (DP), the hydrolysis of extracellular GSH is catalyzed to its constituent amino acids, glutamate, cysteine and glycine. The three amino acids are adsorbed by transporters. Additionally, intestinal epithelial cells can import intact GSH from the lumen via specific plasma membrane transporters.

Cytosolic synthesis of GSH takes place in two ATP-dependent reactions catalyzed by glutamate-cysteine ligase (GCL) and glutathione synthase (GS). The activity of these enzymes is regulated by many pathways, suggesting that the synthesis of GSH must respond to a multitude of environmental conditions. TGFβ1 is one of the major repressors of GCL expression leading to lower levels of GSH [9]; because the cytokine activity is induced by ROS and leads to fibrosis [10,11], this implies that in a pro-fibrogenic environment, the depletion of GSH occurs through multiple mechanisms, either by oxidation or by decreased synthesis [12]. In addition, hypoxia represents an inhibitory signal for GSH synthesis, since it decreases the activity of the two key biosynthetic enzymes GCL and GS [13]. On the other hand, vitamin D induces the expression of GCL and glutathione reductase genes [14], thus increasing the levels of GSH. The GLC gene is induced also by exposure to reactive oxygen species and nitric oxide species [15].

Glutathione exists in reduced (GSH) and oxidized (GSSG) states. In the reduced state, the thiol group of cysteine is able to donate a reducing equivalent (H^+^ + e^−^) to other unstable molecules, such as ROS. In donating an electron, glutathione itself becomes reactive, but readily reacts with another reactive glutathione to form glutathione disulfide (GSSG). Such a reaction is possible due to the relatively high concentration of glutathione in cells (up to 5 mM in the liver). The regeneration of GSH from GSSG is catalyzed by glutathione reductase (GR) in the GSH redox cycle. GSSG reduction occurs at the expense of NADPH, produced by the pentose phosphate pathway (PPP) from glucose oxidation. The intracellular GSH pool, present in millimolar concentrations, is involved in various GSH-dependent reactions. Compartmentalization of GSH within the mitochondria, nucleus or endoplasmic reticulum creates distinct and independently regulated subcellular redox pools.

Glutathione plays a cytoprotective role through several mechanisms. As a reducing agent, it is the main cellular antioxidant agent in the reduction of hydrogen peroxide (H_2_O_2_) and lipid hydroperoxides (LOOH) catalyzed by glutathione peroxidases (GPXs). In the reactions of protection against ROS, the role of glutathione is illustrated by the reduction of peroxides:

2 GSH + R_2_O_2_ → GSSG + 2 ROH

R = H or alkyl group, and by the neutralization of free radicals:

GSH + R → 0.5 GSSG + RH

GSH takes part also in the reduction of protein disulfides (PrSSG) catalyzed by glutaredoxins (GRXs). Moreover, it is employed in the preservation of protein-dithiols by the protein disulfide reductase (glutathione) (EC 1.8.4.2), an enzyme that catalyzes the chemical reaction:

2 glutathione + protein disulfide ↔ glutathione disulfide + protein-dithiol.

This enzyme acts on different substrates, such as insulin, which is inactivated by cleavage of the disulfide bridge, or on protein disulfide, in which the reduction to protein-dithiol restores the catalytic activity, protecting the cell from oxidant stress.

Another important function of glutathione is mediated by its conjugation to several substrates. In the detoxifying defense systems, GSH participates in conjugation reactions catalyzed by glutathione-S-transferases (GSTs). Many heavy metals such as mercury and lead are eliminated as GSH conjugates to prevent their irreversible binding to SH groups of many enzymes, including many membrane ATPase. Subacute exposure to lead results in GSH pool depletion and accumulation of lipid peroxidation products and modifies the activity of the glutathione-related enzymes, such as GR, GST and glucose-6-phosphate dehydrogenase [16]. Many lipophilic xenobiotics are conjugated to GSH to facilitate their excretion or further metabolism. Conjugate compounds are exported from the cell by GSH transporters, which requires ATP for active pumping [17]. In particular, the multidrug resistance-associated proteins (MRP/ABCC) appear to mediate GSH export and homeostasis. The MRP proteins mediate not only GSH efflux, but they also export oxidized glutathione derivatives (e.g., glutathione disulfide (GSSG), S-nitrosoglutathione (GS-NO) and glutathione-metal complexes), as well as other glutathione S-conjugates [18]. Moreover, GSH can be conjugated to proteins in a process called protein S-glutathionylation (PSSG). PSSG protects protein cysteines from being overoxidized to sulfinic and sulfonic species that are not readily regenerated. S-glutathionylation also changes the protein structure and function.

Due to the strong cytoprotective effects of GSH, alterations in GSH homeostasis have been associated with neurodegenerative diseases, AIDS, liver and heart disease, aging, diabetes mellitus and cancer (reviewed in [19,20,21]).

## 3. The Protective Actions of GSH

Glutathione can prevent damage to important cellular components caused by ROS and their derivatives, such as free radicals, peroxides, lipid peroxides, or by organic pollutants and heavy metals. In addition, due to the peculiar reactivity of the –SH group, GSH is involved in several chemical reactions, from disulfide bridge reduction to conjugation to endogenous molecules and xenobiotics. As the pool of the available GSH molecules is fixed, any unexpected increase of its utilization leads to a decrease of the free molecules and impairment of the competing pathways. For example, when GSH is conjugated, it is stolen from the enzymes using the molecule as a cofactor or substrate. This observation is relevant to explain why a deficiency of GSH can occur and can affect pathways involved in severe viral symptoms. Among the many functions of GSH, some are worthy of mention in relation to their impact on the exacerbated inflammation taking place in COVID-19 and in relation to the symptoms developed in the disease.

1. GSH protects cells by neutralizing (i.e., reducing) ROS, which are key signaling molecules that play an important role in the progression of inflammatory disorders. The relationship between ROS production and proinflammatory cytokine activation is well established [22]. An enhanced ROS generation by polymorphonuclear neutrophils at the site of inflammation causes endothelial dysfunction and tissue injury [23].
2. The conjugation of GSH to xenobiotics is particularly abundant; glutathione S-transferase enzymes catalyze GSH conjugation to lipophilic xenobiotics, facilitating the excretion or further metabolism of many drugs. The conjugation process is illustrated by the metabolism of *N*-acetyl-*p*-benzoquinone imine (NAPQI). NAPQI is a reactive metabolite formed by the action of cytochrome P450 on paracetamol (acetaminophen). Glutathione conjugates to NAPQI and the resulting product is excreted [24].
3. Many enzymes use GSH as a cofactor or substrate. For example reactions requiring GSH as a key cofactor are catalyzed by the prostaglandin H synthase, the rate-limiting enzyme in the production of prostaglandins and thromboxane [25], which are essential regulators of vascular function. Moreover, the enzyme leukotriene C(4) synthase conjugates LTA(4) with GSH to form the leukotriene LTC(4), the parent compound of the cysteinyl leukotrienes [26]; these molecules are potent mediators of airway narrowing.
4. GSH is used to synthesize S-nitrosoglutathione (GSNO), an endogenous S-nitrosothiol that plays a critical role in nitric oxide (NO) signaling and is a source of bioavailable NO. The generation of GSNO can serve as a stable NO pool which can properly transduce NO signaling [27]. NO produced by nitric oxide synthase eNOS and nNOS, in the presence of GSH, can effectively modulate vessels and neuronal functions, regulating the blood flow according to the local calcium influx.

As a consequence of the competition between these and many other GSH-consuming pathways, on one hand, the raging inflammation and oxidative stress triggered by the viral infection steals GSH from core functions such as NO-dependent vasodilatation; on the other hand, when other biochemical pathways are consuming GSH, the patient is not protected from an inflammation that can prove fatal.

## 4. The Beneficial Effects of GSH on the Inflammation Driven by the Imbalance of ACE/ACE2

**Figure 2:**
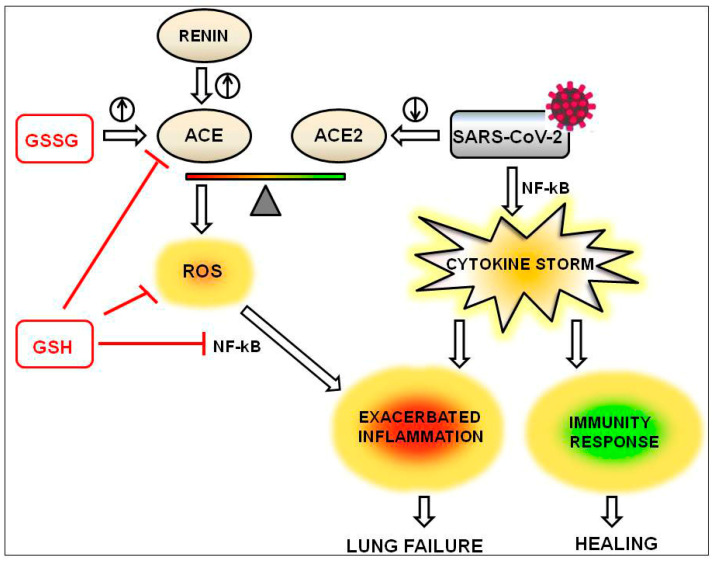
The anti-inflammatory effects of reduced glutathione (GSH) are exerted through the inhibition of ACE activity, decrease of reactive oxygen species (ROS) production and reduction of NF-kB activation (red lines). The balance ACE/ACE2 is shifted toward ACE by the oxidized form of glutathione (GSSG) and by renin (the circled arrows pointing upwards indicate the induction of ACE) and by viral infection (the circled arrow pointing downwards indicates the downregulation of ACE2).

The harmful increase of ANGII can depend on many factors. First, it can be due to the increased renin activity, which is not affected by GSH. Instead, ACE expression and activity are modulated by glutathione; in fact, the oxidized form GSSG shows an activating effect on ACE activity, whereas the reduced GSH provides an inhibitory effect [28]. Finally, the boosted ANGII production can be due to decreased ACE2 expression and activity; this is the case with coronavirus infection, which recognizes ACE2 as its extracellular binding site [29]. Compared to SARS-COV-1, SARS-CoV-2 has about 4-fold higher affinity for ACE2 [30]. Infection of cells by SARS viruses that bind ACE2 results in two effects: inhibition of ACE2 activity and decrease of ACE2 expression in infected cells [29,31,32]. The increased ANGII, through binding to AT1R, activates NADPH oxidases that transfer an electron from NADPH to O_2_ generating several radical species, which can be scavenged by GSH. ROS-mediated oxidation can, in turn, alter gene expression through the induction of signaling cascades or the interaction with transcription factors [33]. Among these factors, a prominent role is played by NF-kB, whose role in inflammation in severe acute respiratory syndrome (SARS) has been demonstrated in both SARS-CoV-infected cultured cells and mice [34]. Drugs that inhibit NF-κB activation lead to a reduction in inflammation and lung pathology. NF-kB is involved in inflammation through multiple mechanisms. In vitro, the viral nucleocapsid (N) protein activates interleukin-6 (IL-6) expression through NF-kB binding at the promoter region of the gene [35]. High levels of IL-6 in the acute stage associated with lung lesions were found in SARS patients [36]. By reducing ROS production, GSH inhibits NF-kB activation and consequently keeps the cytokine storm under control. The effects of GSH are outlined in Figure 2.

The oxidant/antioxidant imbalance is not peculiar to SARS, but it is shared by all inflammatory lung diseases in which the activation of redox-sensitive transcription factors such as NF-kB is reduced by GSH [37]. In an animal model of oxidative stress, NF-κB binding activity was inversely related to liver glutathione and was further suppressed by oral administration of green tea extract [38].

## 5. Conditions Associated with Low GSH

GSH plays a central role in the pathophysiology of human diseases (reviewed in [39]). GSH imbalance is observed in a wide range of pathological conditions including lung infections, HIV, diabetes, cancer and age-related diseases [20,40]. If we consider the conditions clinically associated with severe COVID-19 disease [41,42,43], we find evidence of a perturbed GSH replenishment.

1. Age. Age is a major risk factor for both morbidity and mortality in COVID-19 patients [44,45]. In laboratory animals, the age-related changes in GSH content have been measured in different tissues. Old mice had lower GSH than young mice in several organs. The sharp decrease in the lung is highly remarkable because the sum of aging and air pollution can lead to very low GSH [46]. Similarly to the data obtained in animals, several studies have reported that also in human subjects the concentration of GSH declines with aging [47,48,49,50,51,52].

2. Sex. The severity of COVID-19 is strictly correlated to gender. While men and women have the same prevalence of infection, men with COVID-19 are more at risk for worse outcomes and death, independent of age. In the public data set, the number of men who have died from COVID-19 is 2.4 times that of women (70.3 vs. 29.7%, *p* = 0.016) [53]. Several studies report decreased GSH levels in men compared to women. Research investigating the effect of sex hormones on free radicals and lipid peroxides production found that the erythrocyte glutathione level was lower in healthy men than in healthy women and concluded that the decrease in GSH concentration was not the result of reduced production but probably the result of more rapid utilization against increased oxidative stress triggered by testosterone [54]. Another study reported that neonatal tissues show a gender-dependent modulation of their glutathione metabolism; in fact, in response to oxidative stress, endothelial cells as well as cells derived from tracheal aspirate of baby girls had a greater activity of glutathione reductase compared to tissues derived from baby boys [55]. A study aimed at determining the influence of gender and antioxidant supplementation on exercise-induced oxidative stress reported that, before supplementation, women had higher reduced glutathione and total glutathione compared with men [56].

From data reported in the literature, the difference between sexes in GSH content can be related to hormones or can be ascribed to significant sex-specific differences in drug-metabolizing enzymes.

Estrogens can downregulate the production of ROS triggered by the imbalance of the ACE/ACE2 system; therefore, women have a lower risk of GSH depletion. Indeed, ACE2 expression is modulated by sex hormones. Estrogen modulates the local renin angiotensin system via downregulation of ACE and simultaneous upregulation of ACE2, AT2R and ang (1-7) receptor expression levels [57]. Estradiol directly activates ACE2 expression in different tissues, including differentiated airway epithelial cells [57,58,59]. Moreover serum ACE is significantly lower in female children compared to males after the age 12 [60]. 17beta-estradiol but not other sex hormones such as progesterone and testosterone increases the content of GSH-dependent protein disulfide reductase and protects endothelial cells from oxidative stress [61]. Estradiol production in the peripheral tissues is dependent on the local availability of the precursor DHEA, produced from DHEA sulfate (DHEAS) by steroid-sulfatase. Steroid-sulfatase becomes the limiting enzyme for the synthesis of estrogens in the elderly; whereas the serum DHEAS in young males is much higher than in females, after menopause/andropause, it is very similar. The overexpression of steroid-sulfatase in elderly women can lead to an estrogen production higher than in men even after menopause.

Cortisol also seems to affect glutathione homeostasis. In fact, a study analyzing the administration of anti-inflammatory glucocorticoids in cases of acute respiratory distress syndrome (ARDS) showed that hydrocortisone administration was followed by glutathione depletion and lower glutathione reductase activity in alveolar epithelial type II cells, thus failing to show beneficial effects [62]. Based on these observations, it is possible to infer a role of cortisol in the gender difference of GSH level, as in elderly women, urinary free cortisol excretion is lower than in the elderly men [63].

As for the different sex-related activities of metabolizing enzymes, the specific activity of the catabolic enzyme gamma-glutamyltranspeptidase in female mice was 73% of that in male mice, suggesting that the faster glutathione turnover in males could account for the higher susceptibility to oxidative injury [64]. A time-dependent reduction of hepatic and renal cortical glutathione was observed in both male and female mice following a dose of acetaminophen, but the depletion in male mice was significantly greater than that in the females [65]. This means that even if in basal conditions the GSH levels are similar, after challenges with drugs or pollutants requiring the GSH-dependent detoxification system, the decrease of GSH is more evident in males than in females.

On a large sample of adults from North-Western Italy, vitamin D levels were found significantly higher in women than in men, more elevated in summer than in winter and higher in individuals aged less than 64 years compared to those older than 65 years [66]. As vitamin D induces the expression of GCL and GR genes [14], thus increasing the levels of GSH, these small but significant differences must also be taken into account as preventive factors of severe COVID-19.

3. Diabetes. The correlation between low GSH and diabetes is well established. Diabetes has been widely associated with oxidative damage, increased GSSG/GSH ratio and decreased GSH content in different tissues [67,68]. Decreased GSH is, in most cases, associated with increased activity of NF-kB [69]. A study by Samiec and colleagues showed that the levels of total glutathione and its reduced form were lower in plasma of older subjects and even lower in diabetic patients [70]. In diabetes, the role of GSH as an antioxidant molecule is as critical as its contribution in maintaining the levels of GSNO, the main donor of NO, as discussed above. Insulin resistance is negatively related to the activity of endothelial NO synthase (eNOS), thus creating a link between metabolic and cardiovascular diseases. Lower NO production induces both insulin resistance and hypertension [71]. The increase of insulin sensitivity in muscle, adipocytes and liver depends more on GSNO than on pure NO [72,73].

4. Hypertension. Diabetes and hypertension seem to be the most frequent comorbidities in dying patients [74]. Hypertension may depend on multiple factors; among them it is relevant to cite the activation of the renin-angiotensin system mainly by renin overexpression secondary to low vitamin D, the decreased activity of eNOS and the decreased levels of GSH. As already described for diabetes, the combination low GSH/low NO leads to a higher calcium influx into the vessel wall and in smooth muscle with vasoconstriction, as demonstrated also in animal models [75]. Since in hypertensive patients the low levels of GSH are often accompanied by a decrease of companion enzymes (catalase, GSH-peroxidase and GST) and increased lipid peroxides, the prevailing hypothesis is that the GSH decrease is due to a burst of ROS production, secondary to an inflammatory process [76]. It has been demonstrated that GSH depletion induces chronic oxidative stress and causes hypertension in normal rats. This is accompanied by inactivation and sequestration of NO by ROS, leading to diminished NO bioavailability [77].

5. Obesity. Together with the most common comorbidities, hypertension (56.6%) and diabetes (33.8%), obesity was also found to be closely associated with COVID-19 (41.7%) in patients hospitalized in a US health care system [78]. Many studies suggest that in obese subjects oxidative stress and chronic inflammation are important underlying factors leading to development of many pathologies such as diabetes and cardiovascular diseases. Several studies have reported that in obese patients oxidative stress is associated with diminished glutathione levels [79,80] and decreased GSH/GSSG ratio [81]. In addition, nutritional stress caused by a high fat high carbohydrate diet promotes oxidative stress, as evident by increased lipid peroxidation products, a diminished antioxidant system and decreased glutathione levels [82,83].

6. Pharmacotherapy. Glutathione S-transferase (GST) enzymes catalyze the conjugation of GSH to lipophilic xenobiotics, which include most of the drugs. Acetaminophen (paracetamol) is the best-known drug affecting GSH levels by this mechanism (more than 600 entries in PubMed on this interaction). Many other drugs decrease GSH levels because they induce oxidative stress, for example doxorubicin (adriamycin), antimalarial drugs, chloroquine (CQ), etoposide, opiates, ethanol [84] and antidepressants [85]. The use of CQ deserves special caution because of its chemical properties. In addition to its prooxidant activity leading to GSH depletion [86], chloroquine accumulates into lysosomes leading to their alkalinization and to the impaired uptake of many nutrients from the blood, including transferrin-bound iron. Iron deficiency in the nerve reduces cytochrome C synthesis, respiratory chain activity and ATP synthesis. This toxicity is untreatable and can progress to blindness [87].

Based on these observations, we conclude that many seemingly marginal health problems can concur in creating an individual fragility in the antioxidant defenses, which could be aggravated by the viral infection and could lead to the severe outcome of COVID-19.

## 6. Treatment with GSH and Thiols

Anti-oxidant therapies exert beneficial effects on many diseases characterized by inflammation consequent to impaired redox homeostasis [88,89,90]. In the context of inflammatory diseases, systemic oxidative stress is detected as decreased total free thiol levels (free sulfhydryl groups of cysteine in proteins such as albumin as well as low-molecular-weight free thiols, for example cysteine, glutathione, homocysteine and related species). A recent study has concluded that low molecular mass systemic thiols might play a role in the inflammatory and oxidative stress pathways involved in both chronic obstructive pulmonary disease (COPD) and cardiovascular disease [91]. The levels of systemic free thiols can be influenced by nutritional or therapeutic intervention [92]. For these reasons, many clinical trials have evaluated the efficacy of *N*-acetylcysteine (NAC) administration, and many are still ongoing (714 studies, 349 completed), as well as the effects of GSH supplementation (162 studies, 100 completed) (https://clinicaltrials.gov/).

NAC is both a thiol with antioxidant properties and one of the substrates in GSH biosynthesis. The protective effects of the administration of antioxidant NAC on a murine asthma model was demonstrated in two studies. Treatment with NAC was able to attenuate the diminished ratio GSH/GSSG in animals [93], and the repletion of glutathione pool by NAC counteracted allergen induced airway reactivity/inflammation and restored oxidant-antioxidant balance [94].

In humans, NAC and GSH supplementation has demonstrated its efficacy in several pathologies, and the most interesting results have been obtained in cardiovascular, pulmonary and viral diseases.

NAC has been found effective in acute myocardial infarction (AMI) [95]; indeed, oral NAC supplementation reduced the levels of some inflammatory markers in AMI patients receiving fibrinolytic therapy, and it was judged a therapeutic option for the successful management of these patients.

A study investigating the acute anti-hypertensive effect of anti-oxidant agents in hypertensive subjects and diabetic patients found that the anti-oxidant GSH showed a significant hypotensive effect probably due to the control exerted over the nitric oxide-free radical interaction [96].

In several chronic pulmonary diseases, the correlation between oxidative stress and pathogenesis has been described. The levels of GSH were reported to be markedly decreased in lung fluids and plasma of patients with idiopathic pulmonary fibrosis (IPF). Numerous studies have demonstrated that the administration of various antioxidants is protective against the development of fibrosis in this pathology; among them, some trials aimed at testing the efficacy of GSH precursor, NAC, reported its efficacy in augmenting pulmonary GSH levels and alleviating oxidative stress, although with different results (reviewed in [97]). Many studies found beneficial effects of NAC in COPD patients (reviewed in [98]), although the efficacy of NAC in reducing disease severity was ascribed to NACs mucolytic activity rather than its function as GSH precursor [97]. Alterations in GSH levels and in some GSH-dependent enzymes have also been reported in asthma [97].

The levels of oxidative stress are also critical in the immune response to viruses. During viral infections, an intracellular GSH depletion is mediated by multiple mechanisms and is crucial for viral replication [99]. Although several in vitro and in vivo studies demonstrate that the administration of GSH inhibits viral replication, to date very few clinical trials support the pharmacological use of NAC in respiratory viral infection in vivo (reviewed in [100]). GSH treatment is a promising approach, but high doses of GSH are necessary to achieve a therapeutic efficacy, due to its poor transport into the cells and tissues. GSH delivery could be improved by some derivatives with hydrophobic chains of different length or by I-152, which is a conjugate of NAC and s-acetyl-β-mercaptoethylamine (MEA), which is able to release NAC and MEA and increase GSH content [100].

GSH has been used locally in the treatment of emphysema, where experimental pieces of evidence demonstrated that the oxidative downregulation of the activity of α-1-proteinase inhibitor was curtailed by glutathione, and the authors of the study suggest that this treatment can be considered an option for acute respiratory crises due to COPD [101]. Previous clinical trials of nebulized GSH have demonstrated the bioavailability and safety of up to 600 mg twice daily [101]. As an increased ROS production in COVID-19 is the currently prevailing hypothesis, this approach could be suitable in this case as well.

GSH is one of the more represented molecules in our body: its concentration is 2–5 mM, comparable to very abundant molecular species such as glucose in the blood (5 mM) and intracellular ATP (5–10 mM). If we assume 40 kg of tissue with an average of 2.5 mM GSH (about 750 mg/L), it means that there is 30 g of GSH in the whole body. With a half-life of 48 h, its life is around 10 days; this means that tissue loses 3 g of GSH a day; therefore, the dietary support for full replacement is approximately 1.5 g glutamate, 0.75 g glycine and 1.20 g cysteine (1.63 g if assumed as *N*-acetyl-l-cysteine, NAC) a day. Body GSH concentration may be increased with oral intake of either GSH, or proteins enriched in the amino acid constituents of GSH, or the supplementation of the two limiting amino acids cysteine and glycine, as the body availability of glutamate is usually not limiting. Oral administration of GSH is more expensive than supplements with cysteine and glycine, and its systemic bioavailability may be poor due to degradation in the gut; therefore, its suitability for use on a large population could be limited. Interestingly, a case report study has shown that the repeated use of both 2000 mg of oral administration and intravenous injection of glutathione was effective in relieving the severe respiratory symptoms of COVID-19, showing for the first time the efficacy of this antioxidant therapy for COVID-19 [102].

Dietary supplementation with the glutathione precursors cysteine and glycine, in proper conditions, fully restores glutathione synthesis and concentrations. Some observations support this practical and effective approach to decrease oxidative stress in aging and diseases associated with low GSH concentration [103]. It was demonstrated that elderly subjects had markedly lower red blood cell concentrations of GSH (53%) compared with younger controls. After oral treatment of 14 days with 0.81 mmol cysteine (132 mg NAC)·kg^−1^·d^−1^ and 1.33 mmol glycine (100 mg)·kg^−1^·d^−1^, the elderly reached the GSH concentration of the younger controls [103].

Five clinical trials are at the moment evaluating the effects of supplementation with *N*-acetylcysteine plus glycine (https://clinicaltrials.gov/).

NAC has been largely used in the past as a treatment for bronchitis. A meta-analysis evaluating 13 studies and a total of 4155 people with COPD concluded that the standard dose of 1200 milligrams of *N*-acetylcysteine per day reduces the incidence and severity of flares (known as exacerbations) compared to a placebo [104]. Considering NAC as one of the substrates in GSH biosynthesis, the stoichiometric dose of glycine should be about 1000 mg (exactly 938 mg). This amount roughly corresponds to the daily turnover in healthy people and seems a reasonable dosage free of side effects, either for a preventive or therapeutic approach.

## 7. Conclusions

The outlined overview describes how SARS-CoV-2 can unbalance a high activity of the renin-angiotensin system in the lung via ACE2 downregulation, followed by free radical mediated inflammation, and unveils the protective role of GSH; this biochemical approach to COVID-19 disease opens novel avenues for further investigation aimed at understanding the involved molecular mechanisms.

Several pieces of evidence reported in our biochemical analysis suggest that low levels of GSH could be one of the major causes of the excessive inflammatory response linked to severe COVID-19 symptoms and indicate that increasing body GSH could reduce the number of symptomatic patients. Future clinical studies investigating the levels of GSH in COVID-19 patients may be the starting point to explore this possibility.

## Abbreviations

GSH	glutathione
SARS-CoV-2	severe acute respiratory syndrome coronavirus 2
ACE	angiotensin-converting enzyme
RAS	renin-angiotensin system
ROS	reactive oxygen species
GST	glutathione S-transferase
GPX	glutathione peroxidase
NAPQI	*N*-acetyl-*p*-benzoquinone imine
LT	leukotriene
GSNO	S-nitrosoglutathione
NO	nitric oxide
NOS	nitric oxide synthase
ANGII	angiotensin II
AT1R	angiotensin II receptor type 1
NF-kB	nuclear factor kappa-light-chain-enhancer of activated B cells
IL-6	interleukin-6
CQ	chloroquine
PEDV	porcine epidemic diarrhea virus
NAC	*N*-acetyl-l-cysteine
COPD	chronic obstructive pulmonary disease

## References

[1] Capettini L.S.A., Montecucco F., Mach F., Stergiopulos N., Santos R.A.S., da Silva R.F. (2012). Role of renin-angiotensin system in inflammation, immunity and aging. Curr. Pharm. Des..

[2] Kuba K., Imai Y., Rao S., Gao H., Guo F., Guan B., Huan Y., Yang P., Zhang Y., Deng W. (2005). A crucial role of angiotensin converting enzyme 2 (ACE2) in SARS coronavirus-induced lung injury. Nat. Med..

[3] Ji X., Zhang C., Zhai Y., Zhang Z., Xue Y., Zhang C., Tan G., Niu G. (2020). TWIRLS, an Automated Topic-wise Inference Method Based on Massive Literature, Suggests a Possible Mechanism via ACE2 for the Pathological Changes in the Human Host after Coronavirus Infection. medRxiv.

[4] Hanff T.C., Harhay M.O., Brown T.S., Cohen J.B., Mohareb A.M. (2020). Is There an Association Between COVID-19 Mortality and the Renin-Angiotensin System—A Call for Epidemiologic Investigations. Clin. Infect. Dis..

[5] Vaduganathan M., Vardeny O., Michel T., McMurray J.J.V., Pfeffer M.A., Solomon S.D. (2020). Renin–Angiotensin–Aldosterone System Inhibitors in Patients with Covid-19. N. Engl. J. Med..

[6] Naik E., Dixit V.M. (2011). Mitochondrial reactive oxygen species drive proinflammatory cytokine production. J. Exp. Med..

[7] Zherebiatiev A., Kamyshnyi A. (2016). Expression Levels of Proinflammatory Cytokines and NLRP3 Inflammasome in an Experimental Model of Oxazolone-induced Colitis. Iran. J. Allergy Asthma. Immunol..

[8] Forman H.J., Zhang H., Rinna A. (2009). Glutathione: Overview of its protective roles, measurement, and biosynthesis. Mol. Aspects Med..

[9] Liu R.-M., Vayalil P.K., Ballinger C., Dickinson D.A., Huang W.-T., Wang S., Kavanagh T.J., Matthews Q.L., Postlethwait E.M. (2012). Transforming growth factor β suppresses glutamate-cysteine ligase gene expression and induces oxidative stress in a lung fibrosis model. Free Radic. Biol. Med..

[10] Gorowiec M.R., Borthwick L.A., Parker S.M., Kirby J.A., Saretzki G.C., Fisher A.J. (2012). Free radical generation induces epithelial-to-mesenchymal transition in lung epithelium via a TGF-β1-dependent mechanism. Free Radic. Biol. Med..

[11] Barcellos-Hoff M.H., Dix T.A. (1996). Redox-mediated activation of latent transforming growth factor-beta 1. Mol. Endocrinol..

[12] Liu R.-M., Gaston Pravia K.A. (2010). Oxidative stress and glutathione in TGF-beta-mediated fibrogenesis. Free Radic. Biol. Med..

[13] Jackson R.M., Gupta C. (2010). Hypoxia and kinase activity regulate lung epithelial cell glutathione. Exp. Lung Res..

[14] Lei G.-S., Zhang C., Cheng B.-H., Lee C.-H. (2017). Mechanisms of Action of Vitamin D as Supplemental Therapy for Pneumocystis Pneumonia. Antimicrob. Agents Chemother..

[15] Biolo G., Antonione R., De Cicco M. (2007). Glutathione metabolism in sepsis. Crit. Care Med..

[16] Dobrakowski M., Pawlas N., Hudziec E., Kozłowska A., Mikołajczyk A., Birkner E., Kasperczyk S. (2016). Glutathione, glutathione-related enzymes, and oxidative stress in individuals with subacute occupational exposure to lead. Environ. Toxicol. Pharmacol..

[17] Wang W., Ballatori N. (1998). Endogenous glutathione conjugates: Occurrence and biological functions. Pharmacol. Rev..

[18] Ballatori N., Krance S.M., Marchan R., Hammond C.L. (2009). Plasma membrane glutathione transporters and their roles in cell physiology and pathophysiology. Mol. Aspects Med..

[19] Reid M., Jahoor F. (2001). Glutathione in disease. Curr. Opin. Clin. Nutr. Metab. Care.

[20] Wu G., Fang Y.-Z., Yang S., Lupton J.R., Turner N.D. (2004). Glutathione metabolism and its implications for health. J. Nutr..

[21] Townsend D.M., Tew K.D., Tapiero H. (2003). The importance of glutathione in human disease. Biomed. Pharmacother..

[22] Agita A., Alsagaff M.T. (2017). Inflammation, Immunity, and Hypertension. Acta Med. Indones.

[23] Mittal M., Siddiqui M.R., Tran K., Reddy S.P., Malik A.B. (2014). Reactive oxygen species in inflammation and tissue injury. Antioxid. Redox Signal..

[24] Hedgpeth B., Missall R., Bambaci A., Smolen M., Yavuz S., Cottrell J., Chu T., Chang S.L. (2019). A Review of Bioinformatics Tools to Understand Acetaminophen-Alcohol Interaction. Medicines.

[25] Burch J.W., Services P.T. (1990). Glutathione disulfide production during arachidonic acid oxygenation in human platelets. Prostaglandins.

[26] Lam B.K. (2003). Leukotriene C(4) synthase. Prostaglandins Leukot. Essent. Fatty Acids.

[27] Balazy M., Kaminski P.M., Mao K., Tan J., Wolin M.S. (1998). S-Nitroglutathione, a product of the reaction between peroxynitrite and glutathione that generates nitric oxide. J. Biol. Chem..

[28] Basi Z., Turkoglu V. (2019). In vitro effect of oxidized and reduced glutathione peptides on angiotensin converting enzyme purified from human plasma. J. Chromatogr. B.

[29] Zhang H., Penninger J.M., Li Y., Zhong N., Slutsky A.S. (2020). Angiotensin-converting enzyme 2 (ACE2) as a SARS-CoV-2 receptor: Molecular mechanisms and potential therapeutic target. Intensive. Care Med..

[30] Walls A.C., Park Y.-J., Tortorici M.A., Wall A., McGuire A.T., Veesler D. (2020). Structure, Function, and Antigenicity of the SARS-CoV-2 Spike Glycoprotein. Cell.

[31] Glowacka I., Bertram S., Herzog P., Pfefferle S., Steffen I., Muench M.O., Simmons G., Hofmann H., Kuri T., Weber F. (2010). Differential downregulation of ACE2 by the spike proteins of severe acute respiratory syndrome coronavirus and human coronavirus NL63. J. Virol..

[32] Banu N., Panikar S.S., Leal L.R., Leal A.R. (2020). Protective role of ACE2 and its downregulation in SARS-CoV-2 infection leading to Macrophage Activation Syndrome: Therapeutic implications. Life Sci..

[33] Vajapey R., Rini D., Walston J., Abadir P. (2014). The impact of age-related dysregulation of the angiotensin system on mitochondrial redox balance. Front. Physiol..

[34] DeDiego M.L., Nieto-Torres J.L., Regla-Nava J.A., Jimenez-Guardeno J.M., Fernandez-Delgado R., Fett C., Castano-Rodriguez C., Perlman S., Enjuanes L. (2014). Inhibition of NF- B-Mediated Inflammation in Severe Acute Respiratory Syndrome Coronavirus-Infected Mice Increases Survival. J. Virol..

[35] Zhang X., Wu K., Wang D., Yue X., Song D., Zhu Y., Wu J. (2007). Nucleocapsid protein of SARS-CoV activates interleukin-6 expression through cellular transcription factor NF-κB. Virology.

[36] Henry B.M., de Oliveira M.H.S., Benoit S., Plebani M., Lippi G. (2020). Hematologic, biochemical and immune biomarker abnormalities associated with severe illness and mortality in coronavirus disease 2019 (COVID-19): A meta-analysis. Clin. Chem. Lab. Med..

[37] Rahman I., MacNee W. (2000). Oxidative stress and regulation of glutathione in lung inflammation. Eur. Respir. J..

[38] Park H.J., Lee J.-Y., Chung M.-Y., Park Y.-K., Bower A.M., Koo S.I., Giardina C., Bruno R.S. (2012). Green Tea Extract Suppresses NFκB Activation and Inflammatory Responses in Diet-Induced Obese Rats with Nonalcoholic Steatohepatitis. J. Nutr..

[39] Franco R., Schoneveld O.J., Pappa A., Panayiotidis M.I. (2007). The central role of glutathione in the pathophysiology of human diseases. Arch. Physiol. Biochem..

[40] Teskey G., Abrahem R., Cao R., Gyurjian K., Islamoglu H., Lucero M., Martinez A., Paredes E., Salaiz O., Robinson B. (2018). Glutathione as a Marker for Human Disease. Adv. Clin. Chem..

[41] Du Y., Tu L., Zhu P., Mu M., Wang R., Yang P., Wang X., Hu C., Ping R., Hu P. (2020). Clinical Features of 85 Fatal Cases of COVID-19 from Wuhan. A Retrospective Observational Study. Am. J. Respir. Crit. Care Med..

[42] Guan W.-J., Liang W.-H., Zhao Y., Liang H.-R., Chen Z.-S., Li Y.-M., Liu X.-Q., Chen R.-C., Tang C.-L., Wang T. (2020). Comorbidity and its impact on 1590 patients with COVID-19 in China: A nationwide analysis. Eur. Respir. J..

[43] Wang B., Li R., Lu Z., Huang Y. (2020). Does comorbidity increase the risk of patients with COVID-19: Evidence from meta-analysis. Aging (Albany NY).

[44] Zhou F., Yu T., Du R., Fan G., Liu Y., Liu Z., Xiang J., Wang Y., Song B., Gu X. (2020). Clinical course and risk factors for mortality of adult inpatients with COVID-19 in Wuhan, China: A retrospective cohort study. Lancet.

[45] Palaiodimos L., Kokkinidis D.G., Li W., Karamanis D., Ognibene J., Arora S., Southern W.N., Mantzoros C.S. (2020). Severe obesity, increasing age and male sex are independently associated with worse in-hospital outcomes, and higher in-hospital mortality, in a cohort of patients with COVID-19 in the Bronx, New York. Metab. Clin. Exp..

[46] Amatore D., Celestino I., Brundu S., Galluzzi L., Coluccio P., Checconi P., Magnani M., Palamara A.T., Fraternale A., Nencioni L. (2019). Glutathione increase by the n-butanoyl glutathione derivative (GSH-C4) inhibits viral replication and induces a predominant Th1 immune profile in old mice infected with influenza virus. FASEB Bioadv..

[47] Erden-Inal M., Sunal E., Kanbak G. (2002). Age-related changes in the glutathione redox system. Cell Biochem. Funct..

[48] Matsubara L.S., Machado P.E. (1991). Age-related changes of glutathione content, glutathione reductase and glutathione peroxidase activity of human erythrocytes. Braz. J. Med. Biol. Res..

[49] Loguercio C., Taranto D., Vitale L.M., Beneduce F., Del Vecchio Blanco C. (1996). Effect of liver cirrhosis and age on the glutathione concentration in the plasma, erythrocytes, and gastric mucosa of man. Free Radic. Biol. Med..

[50] Suh J.H., Wang H., Liu R.-M., Liu J., Hagen T.M. (2004). (R)-alpha-lipoic acid reverses the age-related loss in GSH redox status in post-mitotic tissues: Evidence for increased cysteine requirement for GSH synthesis. Arch. Biochem. Biophys..

[51] Lang C.A., Naryshkin S., Schneider D.L., Mills B.J., Lindeman R.D. (1992). Low blood glutathione levels in healthy aging adults. J. Lab. Clin. Med..

[52] Al-Turk W.A., Stohs S.J., el-Rashidy F.H., Othman S. (1987). Changes in glutathione and its metabolizing enzymes in human erythrocytes and lymphocytes with age. J. Pharm. Pharmacol..

[53] Jin J.-M., Bai P., He W., Wu F., Liu X.-F., Han D.-M., Liu S., Yang J.-K. (2020). Gender Differences in Patients With COVID-19: Focus on Severity and Mortality. Front. Public Health.

[54] Dincer Y., Ozen E., Kadioglu P., Hatemi H., Akçay T. (2001). Effect of sex hormones on lipid peroxidation in women with polycystic ovary syndrome, healthy women, and men. Endocr. Res..

[55] Lavoie J.C., Chessex P. (1997). Gender and maturation affect glutathione status in human neonatal tissues. Free Radic. Biol. Med..

[56] Goldfarb A.H., McKenzie M.J., Bloomer R.J. (2007). Gender comparisons of exercise-induced oxidative stress: Influence of antioxidant supplementation. Appl. Physiol. Nutr. Metab..

[57] Bukowska A., Spiller L., Wolke C., Lendeckel U., Weinert S., Hoffmann J., Bornfleth P., Kutschka I., Gardemann A., Isermann B. (2017). Protective regulation of the ACE2/ACE gene expression by estrogen in human atrial tissue from elderly men. Exp. Biol. Med..

[58] Liu J., Ji H., Zheng W., Wu X., Zhu J.J., Arnold A.P., Sandberg K. (2010). Sex differences in renal angiotensin converting enzyme 2 (ACE2) activity are 17β-oestradiol-dependent and sex chromosome-independent. Biol. Sex Differ..

[59] Stelzig K.E., Canepa-Escaro F., Schiliro M., Berdnikovs S., Prakash Y.S., Chiarella S.E. (2020). Estrogen regulates the expression of SARS-CoV-2 receptor ACE2 in differentiated airway epithelial cells. Am. J. Physiol. Lung Cell Mol. Physiol..

[60] Landazuri P., Granobles C., Loango N. (2008). Gender differences in serum angiotensin-converting enzyme activity and blood pressure in children: An observational study. Arq. Bras. Cardiol..

[61] Ejima K., Nanri H., Araki M., Uchida K., Kashimura M., Ikeda M. (1999). 17beta-estradiol induces protein thiol/disulfide oxidoreductases and protects cultured bovine aortic endothelial cells from oxidative stress. Eur. J. Endocrinol..

[62] Walther U.I. (2004). Changes in the glutathione system of lung cell lines after treatment with hydrocortisone. Arch. Toxicol..

[63] Barton R.N., Horan M.A., Weijers J.W., Sakkee A.N., Roberts N.A., van Bezooijen C.F. (1993). Cortisol production rate and the urinary excretion of 17-hydroxycorticosteroids, free cortisol, and 6 beta-hydroxycortisol in healthy elderly men and women. J. Gerontol..

[64] Ma Y. (1998). Sex differences in oxidative damage in ddY mouse kidney treated with a renal carcinogen, iron nitrilotriacetate. Carcinogenesis.

[65] Hu J.J., Lee M.J., Vapiwala M., Reuhl K., Thomas P.E., Yang C.S. (1993). Sex-Related Differences in Mouse Renal Metabolism and Toxicity of Acetaminophen. Toxicol. Appl. Pharmacol..

[66] Basile M., Ciardi L., Crespi I., Saliva E., Bellomo G., Vidali M. (2013). Assessing Serum Concentrations of 25-Hydroxy-Vitamin D in North-Western Italy. J. Frailty Aging.

[67] Yoshida K., Hirokawa J., Tagami S., Kawakami Y., Urata Y., Kondo T. (1995). Weakened cellular scavenging activity against oxidative stress in diabetes mellitus: Regulation of glutathione synthesis and efflux. Diabetologia.

[68] Thornalley P.J., McLellan A.C., Lo T.W.C., Benn J., Sönksen P.H. (1996). Negative Association between Erythrocyte Reduced Glutathione Concentration and Diabetic Complications. Clin. Sci..

[69] Arnalich F., Hernanz A., López-Maderuelo D., de la Fuente M., Arnalich F.M., Andrés-Mateos E., Fernández-Capitán C., Montiel C. (2001). Intracellular glutathione deficiency is associated with enhanced nuclear factor-κB activation in older noninsulin dependent diabetic patients. Free Radic. Res..

[70] Samiec P.S., Drews-Botsch C., Flagg E.W., Kurtz J.C., Sternberg P., Reed R.L., Jones D.P. (1998). Glutathione in human plasma: Decline in association with aging, age-related macular degeneration, and diabetes. Free Radic. Biol. Med..

[71] Petrie J.R., Ueda S., Webb D.J., Elliott H.L., Connell J.M.C. (1996). Endothelial Nitric Oxide Production and Insulin Sensitivity: A Physiological Link With Implications for Pathogenesis of Cardiovascular Disease. Circulation.

[72] McGrowdera D., Ragoobirsingh D., Brown P. (2006). Modulation of glucose uptake in adipose tissue by nitric oxide-generating compounds. J. Biosci..

[73] Fernandes A.B., Guarino M.P., Macedo M.P. (2012). Understanding the in-vivo relevance of *S* -nitrosothiols in insulin action. Can. J. Physiol. Pharmacol..

[74] Fang L., Karakiulakis G., Roth M. (2020). Are patients with hypertension and diabetes mellitus at increased risk for COVID-19 infection?. Lancet Respir. Med..

[75] Vaziri N.D., Wang X.Q., Oveisi F., Rad B. (2000). Induction of oxidative stress by glutathione depletion causes severe hypertension in normal rats. Hypertension.

[76] Guo D., Gu J., Jiang H., Ahmed A., Zhang Z., Gu Y. (2016). Inhibition of pyruvate kinase M2 by reactive oxygen species contributes to the development of pulmonary arterial hypertension. J. Mol. Cell. Cardiol..

[77] Zhou X.J., Vaziri N.D., Wang X.Q., Silva F.G., Laszik Z. (2002). Nitric oxide synthase expression in hypertension induced by inhibition of glutathione synthase. J. Pharmacol. Exp. Ther..

[78] Richardson S., Hirsch J.S., Narasimhan M., Crawford J.M., McGinn T., Davidson K.W., Barnaby D.P., Becker L.B., Chelico J.D., The Northwell COVID-19 Research Consortium (2020). Presenting Characteristics, Comorbidities, and Outcomes Among 5700 Patients Hospitalized With COVID-19 in the New York City Area. JAMA.

[79] Habib S.A., Saad E.A., Elsharkawy A.A., Attia Z.R. (2015). Pro-inflammatory adipocytokines, oxidative stress, insulin, Zn and Cu: Interrelations with obesity in Egyptian non-diabetic obese children and adolescents. Adv. Med. Sci..

[80] Uzun H., Konukoglu D., Gelisgen R., Zengin K., Taskin M. (2007). Plasma protein carbonyl and thiol stress before and after laparoscopic gastric banding in morbidly obese patients. Obes. Surg..

[81] Zamora-Mendoza R., Rosas-Vargas H., Ramos-Cervantes M.T., Garcia-Zuniga P., Perez-Lorenzana H., Mendoza-Lorenzo P., Perez-Ortiz A.C., Estrada-Mena F.J., Miliar-Garcia A., Lara-Padilla E. (2018). Dysregulation of mitochondrial function and biogenesis modulators in adipose tissue of obese children. Int. J. Obes..

[82] Parsanathan R., Jain S.K. (2019). Glutathione deficiency induces epigenetic alterations of vitamin D metabolism genes in the livers of high-fat diet-fed obese mice. Sci. Rep..

[83] Andrich D.E., Melbouci L., Ou Y., Auclair N., Mercier J., Grenier J.-C., Lira F.S., Barreiro L.B., Danialou G., Comtois A.-S. (2019). A Short-Term High-Fat Diet Alters Glutathione Levels and IL-6 Gene Expression in Oxidative Skeletal Muscles of Young Rats. Front. Physiol..

[84] Ponsoda X., Jover R., Gómez-Lechón M.J., Fabra R., Trullenque R., Castell J. (1991). Intracellular glutathione in human hepatocytes incubated with S-adenosyl-L-methionine and GSH-depleting drugs. Toxicology.

[85] Post A., Crochemore C., Uhr M., Holsboer F., Behl C. (2000). Differential induction of NF-kappaB activity and neural cell death by antidepressants in vitro. Eur. J. Neurosci..

[86] Bhattacharyya B., Chatterjee T.K., Ghosh J.J. (1983). Effects of chloroquine on lysosomal enzymes, NADPH-induced lipid peroxidation, and antioxidant enzymes of rat retina. Biochem. Pharmacol..

[87] Tacconelli E., Tumbarello M., Camilli G., Bertagnolio S. (2001). Case report: Retinopathy after malaria prophylaxis with chloroquine. Am. J. Trop. Med. Hyg..

[88] Vera M., Torramade-Moix S., Martin-Rodriguez S., Cases A., Cruzado J.M., Rivera J., Escolar G., Palomo M., Diaz-Ricart M. (2018). Antioxidant and Anti-Inflammatory Strategies Based on the Potentiation of Glutathione Peroxidase Activity Prevent Endothelial Dysfunction in Chronic Kidney Disease. Cell. Physiol. Biochem..

[89] Wu T., Gao Y., Guo X., Zhang M., Gong L. (2018). Blackberry and Blueberry Anthocyanin Supplementation Counteract High-Fat-Diet-Induced Obesity by Alleviating Oxidative Stress and Inflammation and Accelerating Energy Expenditure. Oxid. Med. Cell Longev..

[90] He Y., Yue Y., Zheng X., Zhang K., Chen S., Du Z. (2015). Curcumin, inflammation, and chronic diseases: How are they linked?. Molecules.

[91] Zinellu A., Zinellu E., Sotgiu E., Fois A.G., Paliogiannis P., Scano V., Piras B., Sotgia S., Mangoni A.A., Carru C. (2020). Systemic transsulfuration pathway thiol concentrations in chronic obstructive pulmonary disease patients. Eur. J. Clin. Investig..

[92] Bourgonje A.R., Feelisch M., Faber K.N., Pasch A., Dijkstra G., van Goor H. (2020). Oxidative Stress and Redox-Modulating Therapeutics in Inflammatory Bowel Disease. Trends. Mol. Med..

[93] Song J., Yao L., Shi J., Li J., Xu C. (2020). Protective effects of N-acetylcysteine on a chemical-induced murine model of asthma. J. Asthma.

[94] Nadeem A., Siddiqui N., Alharbi N.O., Alharbi M.M., Imam F., Sayed-Ahmed M.M. (2014). Glutathione modulation during sensitization as well as challenge phase regulates airway reactivity and inflammation in mouse model of allergic asthma. Biochimie.

[95] Wasyanto T., Yasa’ A., Jalaludinsyah A. (2019). Effect of Oral N-Acetylcysteine Supplementation on the Immunity System in Patients with Acute Myocardial Infarction. Acta Med. Indones.

[96] Ceriello A., Giugliano D., Quatraro A., Lefebvre P.J. (1991). Anti-oxidants show an anti-hypertensive effect in diabetic and hypertensive subjects. Clin. Sci..

[97] Janssen-Heininger Y., Reynaert N.L., van der Vliet A., Anathy V. (2020). Endoplasmic reticulum stress and glutathione therapeutics in chronic lung diseases. Redox Biol..

[98] Sanguinetti C.M. (2016). N-acetylcysteine in COPD: Why, how, and when?. Multidiscip. Respir. Med..

[99] Sgarbanti R., Nencioni L., Amatore D., Coluccio P., Fraternale A., Sale P., Mammola C.L., Carpino G., Gaudio E., Magnani M. (2011). Redox regulation of the influenza hemagglutinin maturation process: A new cell-mediated strategy for anti-influenza therapy. Antioxid. Redox Signal..

[100] Checconi P., De Angelis M., Marcocci M.E., Fraternale A., Magnani M., Palamara A.T., Nencioni L. (2020). Redox-Modulating Agents in the Treatment of Viral Infections. Int. J. Mol. Sci..

[101] Lamson D.W., Brignall M.S. (2000). The use of nebulized glutathione in the treatment of emphysema: A case report. Altern. Med. Rev..

[102] Horowitz R.I., Freeman P.R., Bruzzese J. (2020). Efficacy of glutathione therapy in relieving dyspnea associated with COVID-19 pneumonia: A report of 2 cases. Respir. Med. Case Rep..

[103] Sekhar R.V., Patel S.G., Guthikonda A.P., Reid M., Balasubramanyam A., Taffet G.E., Jahoor F. (2011). Deficient synthesis of glutathione underlies oxidative stress in aging and can be corrected by dietary cysteine and glycine supplementation. Am. J. Clin. Nutr..

[104] Cazzola M., Calzetta L., Page C., Jardim J., Chuchalin A.G., Rogliani P., Gabriella Matera M. (2015). Influence of *N*-acetylcysteine on chronic bronchitis or COPD exacerbations: A meta-analysis. Eur. Respir. Rev..

[105] Silvagno F., Vernone A., Pescarmona G.P. COVID-19: Can glutathione (GSH) help to reduce severe symptoms?. https://covid-19.conacyt.mx/jspui/bitstream/1000/2366/1/1101532.pdf.

